# PCA/K-L transformation facial recognition method for vending systems

**DOI:** 10.1371/journal.pone.0336225

**Published:** 2025-12-10

**Authors:** Lu Lin, Yu Sang, Erwei Li

**Affiliations:** 1 Faculty of Business and Technology, University of Cyberjaya, Cyberjaya, Selangor Darul Ehsan, Malaysia,; 2 Deutsche WindGuard (Beijing) Technology Co., Ltd., Beijing, China; Civil Aviation University of China, CHINA

## Abstract

Facial recognition, as an efficient and convenient biometric technology, can be applied to automatic vending systems to achieve functions such as fast checkout and personalized recommendations. To improve the accuracy and processing speed of facial recognition technology, this study designs a facial recognition model for an automatic vending system with an improved principal component analysis method and the Holtling transform. This method reduces the dimensionality of facial features by introducing sample partitioning and histograms into principal component analysis to process facial data. On this basis, the Holtling transform is applied to process the reduced dimensional feature image to obtain the projection value of the face image, making the image easier to recognize. On the renderMe-360 and VoxCeleb2 datasets, the recognition accuracy of the model reached 96.32% and 98.24%, both higher than the comparison methods. The model had an average recognition accuracy of 94.388% in facial recognition from various angles, and showed significant efficiency advantages in feature face construction time and recognition time, especially in high noise conditions, demonstrating strong robustness. Therefore, the proposed model can improve the accuracy of facial recognition, as well as have faster processing speed and noise tolerance, providing new technical value for the intelligent development of automatic vending systems in the future.

## 1. Introduction

With the rapid advancement of technology, traditional retail systems are transitioning towards Automated Vending Systems (AVS). The automatic vending system is mainly composed of multiple fields such as electronic technology, computer technology, network technology, communication technology, and mechanical engineering technology, which can achieve various functions such as automatic vending, inventory management, and goods supply [1]. As an unmanned, 24-hour self-service device, the vending system can not only be widely used in large shopping malls, stations, airports, hospitals, schools and other public places, but also save manpower and material resources [2]. The vending system, with its innovative technology and flexible application, is gradually becoming the focus of the new retail field [3,4]. However, vending systems still face issues such as theft and inaccurate user authentication in practical applications, which affects the security and user experience of the vending system [5]. How to improve the security and user experience of vending systems has become an urgent issue that needs to be addressed.

There is currently a lot of research in the field of automatic vending systems. C. Liu et al. proposed a multi category product recognition method for unmanned vending systems, which used manifold learning to identify the shapes between different products, constructed multi granularity labels to constrain the products, and then referenced a hierarchical label object detection network to capture the multi granularity features of the products. It effectively explored the potential similarities between multiple types of products and performs well in practical applications [6]. M. Grzegorowski et al. proposed a feature extraction framework to develop replenishment plans for AVS. This scheme adopts the latest machine learning methods and introduces survival analysis indicators, effectively dealing with data uncertainty and cold start problems that occur when AVS are out of stock. In practical applications, it can automatically replenish according to demand [7]. N. Ivanov et al. designed a security model with vending systems to protect AVS from network attacks during payment. This model introduced a multi signature transaction token to replace the application programming interface, and switched the network interface in the model to the device channel while accessing the guidance service. It could improve the safety performance of AVS and had portability and scalability [8]. A. Sharif et al. designed ultra-high frequency RFID tags based on inkjet printing for fruit sales in AVS. This label consists of dipole shaped bands and eye shaped rectangular nested slots to alleviate the capacitance effect on the surface of the fruit. This method can reduce the surface loss resistance of fruits and match well with the product recognition model of AVS, improving the sorting and selling efficiency of AVS for fruits [9]. L. Gan et al. designed an AVS with radar gesture recognition, which used exponential weighted averaging to fit the radar background echo for real-time gesture recognition. The average recognition rate of user gestures by the system was 95.9%, and it improved the sales rate and transaction speed of AVS [10].

With the rapid development of biometric technology, Facial Recognition (FR) technology has been widely used in scientific research due to its non-contact, convenient, and intuitive characteristics. A. Atzori et al. proposed a novel low resolution row face recognition method that converted high-resolution images into low resolution images and combined them to train a face recognition model. The facial images generated by this method were more realistic than those generated by other methods, and had a significant effect on improving demographic bias [11]. A. M. Rodriguez et al. introduced a multi task interpretable quality network into facial recognition models and designed a quality pairing protocol to address the issue of low efficiency in identifying suspects in surveillance footage. This model had good universality on different video datasets, improving the efficiency of identifying suspects from surveillance footage [12]. W. Gao et al. Constructed a facial recognition protocol to improve the computational performance of intelligent security systems during the recognition phase. This protocol introduced matrix into user data, and used edge computing to realize rapid response of large-scale face recognition. It could effectively improve the efficiency of facial recognition in intelligent security systems [13]. H. Li et al. proposed an unconstrained facial expression recognition model with reference free network element learning to explore information in facial expressions. This model introduced constraint conditions to overcome the problem of no basic information in the process of element removal learning, and decomposed unconstrained facial images into expression elements and neutral facial features, reducing the interference of irrelevant information. This model could improve the classification performance of facial expressions and significantly enhanced the robustness and generalization performance of recognition models [14]. Y. Gu et al. proposed a deep learning model with multi-source learning to address the issue of computers being unable to recognize human emotions. This model effectively utilized autocorrelation, demonstrating notable efficacy in this domain. These findings corroborate the evident superiority of multimodal over single modal [15].

In summary, FR technology is widely applied in various fields. There are also many studies on AVS, and introducing FR technology into AVS (AVS-FR) can not only improve system security, but also provide personalized services for users. Although FR technology has many advantages, it still faces the problem of low recognition accuracy and processing speed in complex environments. Based on this, this study proposes an AVS face recognition model based on Improved Principal Component Analysis/Karhunen Lo è ve Transform (IPCA/K-L) to enhance the AVS for face recognition in complex environments, adapt to diverse needs in complex environments, and provide new technical parameters for the intelligent development of AVS.

The innovation of the research lies in the proposal of an improved PCA. (1) Establish a collaborative optimization mechanism for dynamic blocking strategies. By adaptively adjusting the granularity of blocks based on facial structural features, enhance the classifier’s ability to capture local key features and improve recognition accuracy. (2) Achieve local histogram equalization, perform independent histogram equalization processing within each block to eliminate lighting interference, thereby enhancing the contrast and recognizability of the image. (3) Achieve global feature dimension compression. By collaboratively optimizing the covariance matrix, extract the principal components across blocks, further enhancing the recognition rate under low-light conditions and reducing the error recognition rate. (4) The hierarchical cascaded processing flow was completed, enhancing the anti-noise capability in dynamic environments, improving the recognition efficiency and accuracy, and demonstrating the robustness and effectiveness of the improved PCA in complex scenarios. (5) A feature compression framework integrating the advantages of multiple technologies has been constructed, combining local and global information to enhance the overall algorithm performance and adapt to various application scenarios.

## 2. Methods and materials

This section first introduces the design of a facial feature extraction and recognition scheme based on improved PCA. Secondly, the construction process of the AVS-FR based on IPCA/K-L transformation is described.

### 2.1 Design of facial feature extraction and recognition scheme with improved PCA

FR technology in AVS confirms individual identity by analyzing and comparing feature information in facial images [16]. However, it still faces some challenges in practical applications, such as accurate user authentication, single payment methods, and lack of personalized recommendation functions, which affect the user experience. How to improve the intelligence level of AVS, while maintaining high recognition accuracy, processing speed, and data security in complex environments, has become a current problem to be solved. Principal Component Analysis (PCA) can decline the dimensionality of original features while minimizing the loss of information [17]. First, by calculating the average face of the sample data, the difference between each sample and the average face is obtained, and then the covariance matrix is constructed to describe the linear relationship between the features. Then, through eigenvalue decomposition of the covariance matrix, the eigenvectors and corresponding eigenvalues are identified, and the principal component with the largest variance is screened out. Finally, the original data is projected into the new feature space according to the selected principal component, thereby reducing the data dimension while preserving as much information as possible. However, PCA has lower accuracy when dealing with FR tasks in complex environments. The pseudocode for PCA is as follows:

Function PCA(InputData):
// 1. Calculate the mean face of the samples
MeanFace = CalculateMean(InputData)
// 2. Calculate the difference between each sample and the mean face
DifferenceFaces = InputData – MeanFace
// 3. Construct the covariance matrix
CovarianceMatrix = CalculateCovariance(DifferenceFaces)
// 4. Compute eigenvalues and eigenvectors
EigenValues, EigenVectors = EigenDecomposition(CovarianceMatrix)
// 5. Sort eigenvalues and corresponding eigenvectors
SortedEigenValues, SortedEigenVectors = SortEigenValuesAndVectors(EigenValues, EigenVectors)
// 6. Select the top k principal components
PrincipalComponents = SelectPrincipalComponents(SortedEigenVectors, k)// k is the number of desired components
// 7. Project the original data onto the principal component space
ProjectedData = Project(InputData, PrincipalComponents)
return ProjectedData

Based on this, this study proposes using IPCA to extract and recognize facial features, to improve the performance of FR systems in complex environments. The pseudocode for IPCA is as follows:

Function IPCA(InputData):
// 1. Partition the sample data
PartitionedSamples = PartitionSamples(InputData)
// 2. Perform histogram equalization on each sub-sample
for each sample in PartitionedSamples:
sample = HistogramEqualization(sample)
// 3. Calculate the mean face for each sub-sample
MeanFaces = CalculateMean(PartitionedSamples)
// 4. Calculate the difference between each sub-sample and the corresponding mean face
DifferenceFaces = PartitionedSamples – MeanFaces
// 5. Construct the covariance matrix
CovarianceMatrix = CalculateCovariance(DifferenceFaces)
// 6. Compute eigenvalues and eigenvectors
EigenValues, EigenVectors = EigenDecomposition(CovarianceMatrix)
// 7. Sort eigenvalues and corresponding eigenvectors
SortedEigenValues, SortedEigenVectors = SortEigenValuesAndVectors(EigenValues, EigenVectors)
// 8. Select the top k significant principal components
PrincipalComponents = SelectPrincipalComponents(SortedEigenVectors, k)// k is the number of desired components
// 9. Project the original data onto the principal component space
ProjectedData = Project(InputData, PrincipalComponents)
return ProjectedData

**Fig 1:**
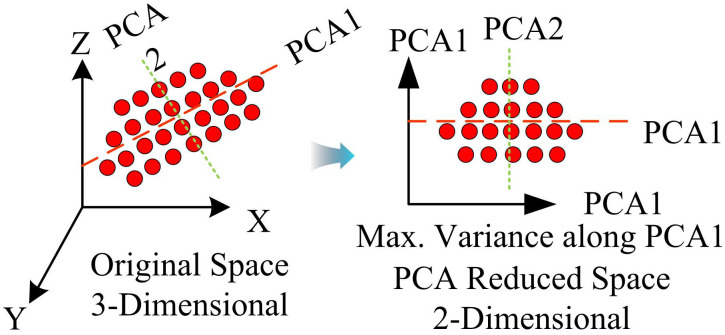
The IPCA dimensionality reduction technique.

The dimensionality reduction method of IPCA is shown in Fig 1.

**Fig 2:**
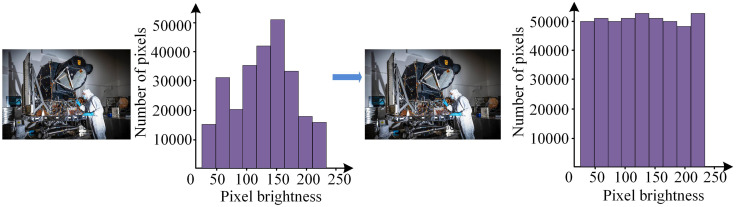
Comparison of image samples before and after histogram equalization (Source from: https://landsat.gsfc.nasa.gov/multimedia/graphics-library/landsat-9-graphics/).

In Fig 1, the dimensionality reduction process in IPCA effectively transforms the original three-dimensional feature space into a two-dimensional space, thus simplifying the complexity of the data and preserving key information. Under this framework, the number of features is reduced from 28 to 20, ensuring that the identification efficiency is improved while the loss of information is minimized. By rearranging the features, the relationship between the distribution of data points and the features can be more clearly reflected in the two-dimensional space. By making clever linear transformations of the raw data, IPCA is able to focus on the most representative features, thereby optimizing subsequent recognition tasks, reducing the computational burden and improving the processing speed and accuracy of the algorithm. This dimensionality reduction strategy not only helps reduce redundant information, but also helps model performance in complex environments, significantly enhancing the robustness of face recognition applications. The horizontal axes PCA1 and vertical axes PCA2 in Fig 1 represent the two directions in which the data changes the most, which results in the first component having the largest variance in the data, followed by the second component, and so on, ensuring that all components are orthogonal to each other. To partition samples with similar features (expressions, angles, lighting) into a matrix, this study adopts a block based approach to partition them. After partitioning, the facial samples shows significant consistency in expressions, facial angles, and environmental lighting. Based on this, the distribution of facial sample data conforms to Gaussian distribution and can improve the efficiency and ability of the algorithm in recognizing sample data [18]. The steps for segmenting facial data samples are as follows: Firstly, the initially set training matrix A is divided into several subset modules $A_{1}$, $A_{2}$... $A_{m}$ The segmented images are subjected to histogram equalization, which enhances the sensitivity and contrast of facial images to highlight sample features [19]. The image effects before and after optimizing the facial dataset using histogram method are shown in Fig 2.

In Fig 2, after histogram equalization optimization, not only did the contrast of the face data in the segmented samples improve, but also the uniqueness of each part of the face image was enhanced [20]. After extracting facial features from the above steps, IPCA is used to extract features from the training samples and recognize them. The size of each facial sample data is m×n dimension, where m is the number of categories in the training set where facial features are collected, and n is the number of samples for each category. The average face and difference face of the sample images are calculated, and all features are decentralized [21]. The formula for calculating the average face is shown in equation (1).


$$\phi =\frac{\text{1}}{mn}\sum_{i=1}^{m}\sum_{j=1}^{n}x_{ij}$$ (1)

In equation (1), X is the data matrix of the original sample; ϕ is the mean vector; $X_{ij}$ is the vectorized value of the j -th sample image of class i. The calculation formula for facial differentiation is shown in equation (2).


$$d_{ij}=x_{ij}-\phi ,i=1,2,...m;j=1,2,...n;$$ (2)

In equation (2), $d_{ij}$ is the difference face vector, representing the difference between each sample image and the average face. At this point, a covariance matrix C can be constructed for the face data image, as shown in equation (3).


$$C=\frac{\text{1}}{mn}AA^{T}$$ (3)

In equation (3), C is the covariance matrix used to describe the linear relationship between features in the dataset. A is the difference face matrix, composed of all difference face vectors; $A^{T}$ is the transpose of the difference face matrix; T is the transpose symbol of the matrix. Due to the complex and multi-step process of directly solving C, the singular value decomposition theorem is used to calculate it. Equation (4) shows the calculation formula [22].


$$u_{i}=\frac{\text{1}}{\sqrt{\lambda_{i}}}Av_{i}$$ (4)

In equation (4), $v_{i}$ is the eigenvector of matrix $AA^{T}$ corresponding to $\lambda_{i}$; $\lambda_{i}$ is the i -th non-zero eigenvalue of matrix $AA^{T}$; $u_{i}$ is the orthogonal normalized eigenvector of matrix $AA^{T}$. On the basis of dimensionality reduction of facial features, based on the influence of feature values on facial features, the top p feature values with the greatest impact on facial features and their feature vectors are selected, and their expressions are shown in equation (5).


$$\frac{\sum_{i=0}^{p}\lambda_{i}}{\sum_{i=0}^{s}\lambda_{i}}\geq \alpha$$ (5)

In equation (5), p is the number of features that have a significant impact on facial features; s is the feature vector value; α is the feature value, usually ranging from 90% to 99%. By filtering, the spatial matrix of the special class face $\omega =\left[u_{1},u_{2},u_{3}\ldots ,u_{p}\right]$ can be obtained. Based on this, the original m×n -dimensional image has been transformed into p -dimensional image features. The image features are classified, and the classification expression is shown in equation (6).


$$y_{ij}=\omega^{T}x_{ij}$$ (6)

In equation (6), $y_{ij}$ is the vectorized image matrix; $\omega^{T}$ is the spatial matrix of the feature face; $x_{ij}$ is the feature vector of the original face image. The vectorized image matrix can be evaluated by a cosine classifier to determine the similarity between two vector matrices. Equation (7) shows the calculation formula.


$$\cos\left(\theta \right)=\frac{y_{ij}y}{\left|y_{ij}\right|\left|y\right|}$$ (7)

**Fig 3:**
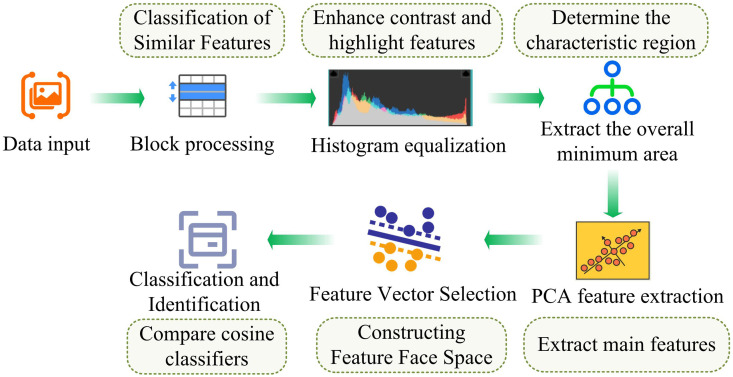
Process of facial feature extraction and recognition method based on ipca algorithm.

In equation (7), cos(θ) is cosine similarity, used to represent the similarity between two feature vectors; y is the feature vector of the test sample; $y_{ij}$ is the feature vector of the training sample. The process of facial feature extraction and recognition based on IPCA is shown in Fig 3. The IPCA process in Fig 3 consists of several key steps. Firstly, the original face image data is processed by sample partitioning and histogram equalization. Then, the mean face and difference face of the partitioned samples are calculated, and the corresponding covariance matrix is constructed. Then, the covariance matrix is calculated by singular value decomposition (SVD), from which the main eigenvectors and eigenvalues are extracted, and the first k most representative features are selected according to the size of the eigenvalues to reduce the dimension. Finally, the original data is projected into the new feature space to form the feature representation after dimensionality reduction. This process not only retains the main information of the data, but also optimizes the face recognition performance in complex environments, effectively improving the accuracy and processing efficiency of the system.

### 2.2 AVS-FR with IPCA/K-L transform

**Fig 4:**
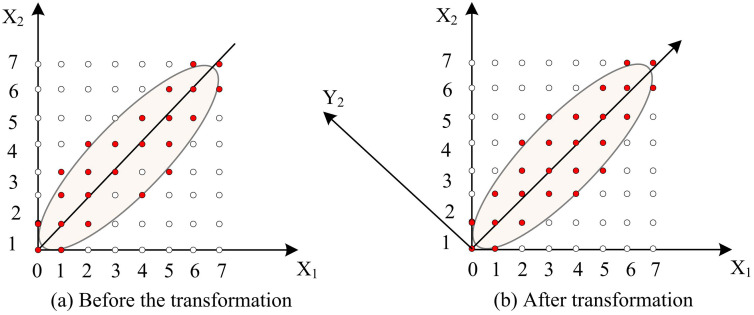
Schematic diagram of K-L variation image block.

By using IPCA to extract features from facial image datasets in the previous section, although the improved algorithm can reduce the dimensionality of images in FR to a certain extent, it has certain limitations when facing images with complex nonlinear features in AVS. AVS typically requires efficient and accurate identification of customers to ensure the security and smooth progress of transactions. Therefore, facial recognition technology is particularly important for rapid response and high accuracy in recognition. K-L transform is a linear transformation method that transforms data from the original space to a new feature space [23]. The K-L transform projects the data onto a new set of coordinate axes, minimizing the correlation between the data on each coordinate axis. The advantage of doing so is that it can reduce redundant information in the data and extract the main features from the data. Based on this, this study introduces the K-L transform into the IPCA algorithm and constructs an AVS-FR model based on the IPCA/K-L transform to address the limitations of traditional recognition methods in complex environments and ensure minimal information loss during dimensionality reduction. Taking image blocks as an example, Fig 4 shows the schematic diagram of K-L transformation.

Fig 4 shows the image blocks before and after K-L transformation. The image pixels have strong correlation in the spatial domain, and the energy distribution is relatively uniform. Secondly, after orthogonal transformation, the energy of the image blocks is concentrated on a few coordinate axes, and the correlation between the transformation coefficients is approximately statistically independent [24]. Firstly, the images in the test and training sets are normalized to obtain the sequence of N×N, which can be continuously formed into an $N^{2}$ -dimensional vector value. Therefore, the image is regarded as a point in the $N^{2}$ -dimensional space and can be described by a low dimensional subspace through K-L transformation. At this point, the generation matrix of K-L transformation is calculated to obtain the value and vector of the input face image itself. Assuming that the face image library N contains images represented by vectors $X_{1},X_{2},\ldots X_{N}$, the mean difference of these images is projected onto this space to obtain the projection vector of a character $Y_{1},Y_{2},\ldots Y_{N}$, and the calculation formula is shown in equation (8).


$$y_{\mu }=(\mu_{j}{)}^{T}X_{j}^{{}^{\prime }},j=1,2,...,M$$ (8)

In equation (8), $y_{\mu }$ is the projection vector, representing the projection of the image in the feature space; ${\left(u_{j}\right)}^{T}$ is the feature vector matrix; $X_{i}^{\prime }$ is the vector representation of the mean difference image. For the face image I to be recognized, the projection vector of its difference from k is calculated using the formula (9).


$$p_{j}=(\mu_{j}{)}^{T}(I_{i}-X_{ave}),j=1,2,...,M$$ (9)

In equation (9), $I_{i}$ is the vector representation of the i -th image; $X_{ave}$ is the average facial image; $p_{j}$ is the projection difference of the image. To further compress the image’s own vector and reduce the computational complexity of the algorithm, this study sorted the size of the image feature vectors and ignored the self vectors corresponding to smaller self values [25]. The compressed feature vector set is shown in equation (10).


$$\bar{U}=[u_{1},u_{2},\ldots ,u_{M}{]}^{T}$$ (10)

In equation (10), $\bar{U}$ is the self vector; $u_{M}$ is the vector matrix; the eigenvalues of the self vectors are sorted in descending order, and the first Z−1 feature sector is retained, where Z is the number of training image categories. Unlike the previous fixed discarding of some self vectors, this process ensures that the information contained in the remaining self vectors is greater than a certain threshold (e). e value is usually 0.9, and the calculation formula is shown in equation (11).


$$e_{i}=\frac{\sum_{j=1}^{i}\lambda_{j}}{\sum_{j=1}^{k}\lambda_{j}}$$ (11)

In equation (11), $\lambda_{j}$ is the eigenvalue of the j -th figure; k is the vector dimension; $e_{i}$ is the information dimension of the i -th figure. Selecting an appropriate distance function from AVS-FR can reduce misidentification, thereby ensuring that AVS can quickly and accurately recognize user information when facing different users. The difference between the absolute values of the pixels of the facial figure is added up as the summation criterion, and the distance function formula is shown in equation (12).


$$L_{1}(x,y)=\sum_{i=1}^{k}\left|x_{i}-y_{i}\right|$$ (12)

**Fig 5:**
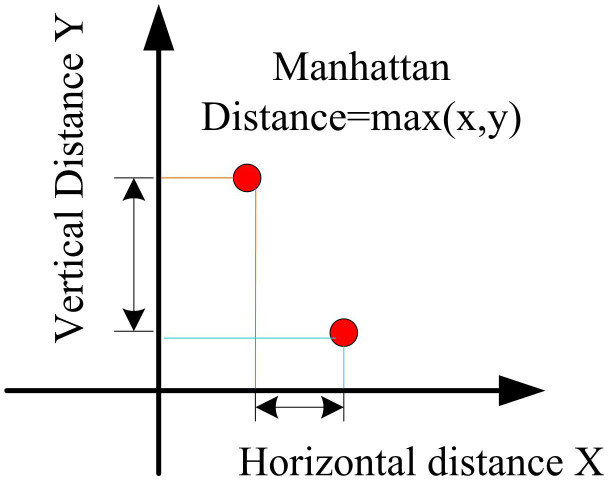
Schematic diagram of K-L variation image block.

In equation (12), $L_{1}(x,y)$ is the L1 distance, which is also the Manhattan distance; $x_{i}$ is the i -th component of the image vector on the x -axis; $y_{i}$ is the i -th component of the image vector on the y -axis. The Manhattan distance only requires addition and subtraction, making the computer more cost-effective in a large number of calculations and eliminating errors caused by taking approximations in the square root process [26]. The schematic diagram is shown in Fig 5.

The L2 normal form distance, also known as Euclidean distance, is obtained by adding the squared difference between the pixels of the graph to the value obtained from the Manhattan distance function. Its calculation formula is shown in equation (13).


$$L_{2}(x,y)=\sum_{i=1}^{k}{\left(x_{i}-y_{i}\right)}^{2}$$ (13)

In equation (13), $L_{2}(x,y)$ is the Euclidean distance value. By calculating the average of different training modes and comparing the distances, each category only needs to be compared once, thereby reducing the computational complexity. The distance expression between samples X and I is shown in equation (14).


$$d_{i}(x)=\sqrt{{\left(x-\mu_{i}\right)}^{T}(x-\mu_{i})}$$ (14)

In equation (14), $d_{i}(x)$ is the distance between the average vectors of sample X and sample I; $\mu_{i}$ is the average value of all samples in class i; x is the vector of the current sample. In the test set of this algorithm, training and recognition are carried out in two stages. In the first stage, to project all information into a specific subspace, the N dimensional vector $Y_{i}\left(i=1,2\cdots N\right)$ needs to be obtained. The expression for the distance threshold is shown in equation (15).


$$\theta_{c}=\frac{\text{1}}{\text{2}}\max\{Y_{j}-Y_{k}\}\;(j,k=1,2,\ldots ,N)$$ (15)

**Fig 6:**
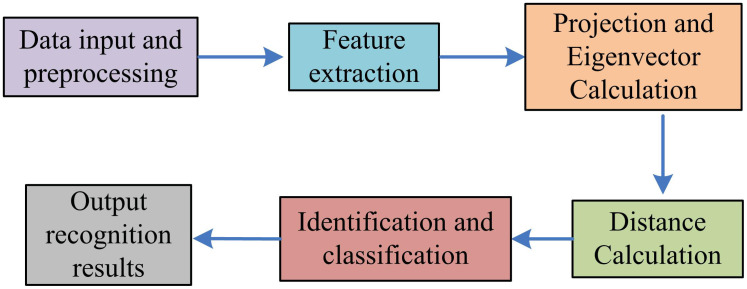
Schematic diagram of K-L variation image block.

In equation (15), $\theta_{c}$ is the distance threshold; $Y_{j}$ and $Y_{k}$ are the projection vectors of different facial shapes; 11 is the two norm of the vectors. The flowchart of the AVS-FR model with IPCA/K-L transformation is shown in Fig 6. The model first normalizes the input facial image dataset, converts it into a vector representation, and extracts features through the IPCA algorithm. The reduced dimensional feature images are then subjected to K-L transformation to obtain the projection values of the facial images. The feature vectors are sorted again, and the most influential features are selected for recognition. Finally, Manhattan distance and Euclidean distance are used to calculate the similarity between the test image and the training image to determine the image category, and threshold judgment is used to ensure the accuracy of recognition and output the recognition result.

Although PCA and K-L transformation are closely related in mathematical principles, in the second stage of the research, there are obvious differences in their scopes of action and optimization objectives. In terms of covariance estimation optimization, PCA only uses the global covariance matrix to extract the principal component directions, ignoring the separability between categories [27]. In the feature space after dimensionality reduction, the K-L transformation is combined with the inter-class adaptive covariance matrix to optimize the feature distribution, such as Formula (8) – Formula (11), and the inter-class differences are enhanced by dynamically adjusting the feature weights. The process of dynamically adjusting feature weights first calculates the performance of each type of sample in the feature dimension, including the contribution of each feature to the discrimination between categories. Based on this information, the model can assign different weights to each feature, highlighting those that have a significant impact on classification while reducing the weights of features that have a smaller impact on classification or contain redundant information [28]. In terms of feature whitening and de-correlation, PCA only decentralizes through orthogonal transformation, and there are still implicit correlations among feature dimensions [29]. The K-L transformation introduces a feature whitening operation, forcing the variance of the feature dimension to be 1 and the covariance to be 0, eliminating redundant correlations and making the Manhattan distance more robust [30]. The specific form is as shown in formulas (12)-(15). Finally, in the information-retention mechanism where energy is concentrated, the principal component selection of PCA is based on the maximization of global variance, which may result in the loss of local highly discriminative features. The K-L transformation retains the local statistical characteristics of different categories and enhances the adaptability to complex nonlinear changes through energy redistribution.

## 3. Results

### 3.1 Validity verification of facial feature extraction and recognition scheme based on IPCA

To verify the effectiveness of the research institute’s design scheme, this study used two different facial datasets to detect the scheme, namely VoxCeleb2 released by Oxford University in 2018 and RenderMe-360 released by Shanghai Artificial Intelligence Laboratory in 2023. RenderMe-360 is a large-scale multi view high-definition facial video dataset, which includes diverse facial expressions, rich fine-grained hairstyles and colors, as well as phonetically balanced speech videos; VoxCeleb2 is a large-scale speaker recognition dataset automatically obtained from open source media, which contains over 100000 facial data information. In this study, the training network architecture of IPCA/K-L transformation model adopts the convolutional neural network (CNN) structure. The architecture includes multiple convolution layers and pooling layers to extract local features of the image, and then IPCA module is introduced for feature dimensionality reduction to retain the main information and reduce the complexity of the data. Then, the K-L transform is applied to further optimize the feature representation and reduce the redundant information. Finally, the extracted features are mapped to the output layer through the full connection layer and classified using the Softmax activation function to achieve efficient and accurate face recognition. The software and hardware environment and initial parameter settings used in the experiment are shown in Table 1.

**Table 1:** Experimental software and hardware configuration and algorithm initialization parameter settings.

Hardware		Algorithm initialization parameter setting table	
Project	Configure	The parameter name	Value
Processor	Intel Core i7-10700K	PCA/K-L dimension	50
Internal storage	16 GBDDR4 RAM	Maximum iteration times	100
Graphics card	NVIDIA GeForce GTX3060	Convergence threshold	0.001
Storage	512 GB SSD	Learning rate	0.01
Camera	Logitech HD Pro Webcam C920	Image size	128 × 128
Software		Feature extraction method	IPCA
Operating system	Windows 1064-bit	Data preprocessing method	Normalization
Programming language	Python 3.8	Training set ratio	80%
The main library	OpenCV, NumPy, scikit-learn	Test set proportions	20%
Exploitation environment	Jupyter Notebook/PyCharm	/	/

To verify the effect of the proposed method in the research, the performance of PCA and PCA + K-L was compared through ablation in the study. The experiment compared the performance differences between the two versions under the same hardware environment with a fixed feature dimension of 50. The specific results are shown in Table 2.

**Table 2:** Ablation experiments of research methods.

Indicator	RenderMe-360		VoxCeleb2	
	PCA	PCA + K-L	PCA	PCA + K-L
Recognition accuracy rate (%)	90.8	96.32	91	98.24
Feature construction time (seconds)	120	125	250	260
Recognition delay (seconds)	1.5	1.7	2	2.2
L1 distance misjudgment rate (%)	8.2	3.5	6.8	1.2
Noise robustness (5dB)	72.1	84.7	75.3	87.9

The results in Table 2 show that, compared with the method using only PCA, the model performance is significantly optimized after introducing the K-L transformation. On the RenderMe-360 dataset, the recognition accuracy rate increased from 90.8% to 96.32%, and on VoxCeleb2, it increased from 91.0% to 98.24%, verifying the enhancement effect of K-L transformation on the classification boundary through feature whitening and energy redistribution. Although the feature construction time and recognition delay increased by approximately 4.2% (5 seconds) and 13.3% (0.2 seconds) respectively due to additional transformation steps, the L1 distance misjudgment rate decreased by 57.3% (RenderM-360) and 82.4% (VoxCeleb2), and the robustness under 5dB high noise improved by 17.5% and 16.8%. It indicates that the K-L transformation achieves a balanced optimization of computational efficiency and recognition accuracy in complex scenarios by eliminating feature redundancy correlations and dynamically adjusting statistical weights, which meets the core requirements of the vending system for high security and low misjudgment.

**Fig 7:**
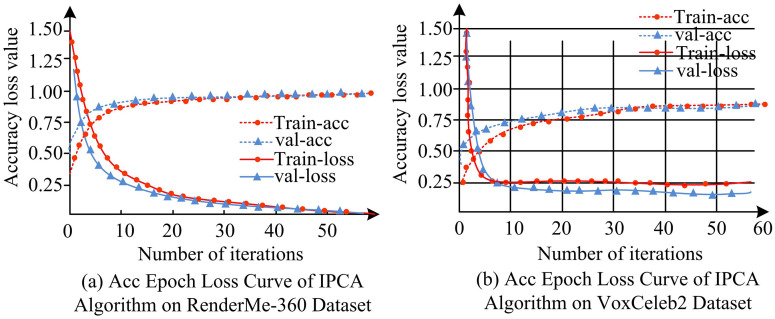
Acc epoch loss curve of IPCA algorithm.

Fig 7 shows the accuracy iteration loss curve of IPAC on two datasets, Acc-Epoch-Loss.

**Fig 8:**
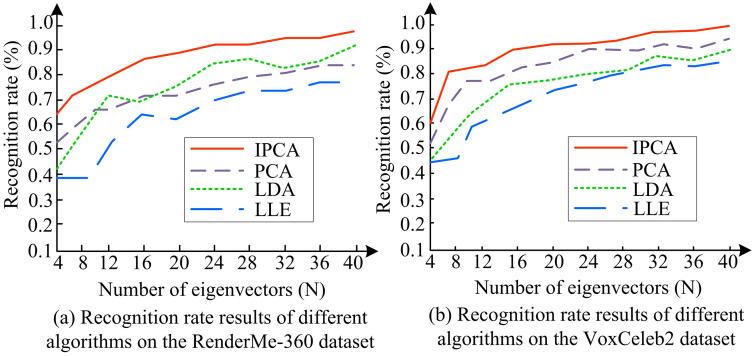
The recognition accuracy of IPCA, PCA, LDA, and LLE with different numbers of feature vectors.

In Fig 7(a), the loss value of IPCA tended to 3% after 60 iterations, with an average accuracy of 97%; In Fig 7(b), the loss value was 23% and the accuracy was 84% after 50 iterations; And the algorithm terminated the iteration in a timely manner at the appropriate position. IPCA effectively captured feature information in images, reduced the loss and neglect of target details, and was more accurate for portrait recognition collected in AVS, reducing the quantity of overfitting occurrences in the algorithm. To verify the accuracy of IPCA in FR in AVS, different numbers of feature vectors were set to validate the recognition accuracy of IPCA, PCA, Linear Discriminant Analysis (LDA), and Locally Linear Embedding (LLE) algorithms [31,32]. Fig 8 shows the experimental outcomes.

In Fig 8(a), the recognition rates of IPCA, PCA, LDA, and LLE all increased with the increase of feature quantity, and the overall recognition rate of IPCA was higher than the other three algorithms. When the number of features was 40, the recognition rates of IPCA, PCA, LDA, and LLE were 96.32%, 84.26%, 90.34%, and 77.49%, respectively, indicating that IPCA had a higher recognition accuracy. This indicated that IPCA could effectively utilize feature information to optimize the facial recognition. In Fig 8(b), IPCA showed a significant increase in recognition accuracy from 60% to 81% as the number of features increased from 4 to 8, while other algorithms showed slower growth. When the number of feature vectors was 40, the recognition rates of IPCA, PCA, LDA, and LLE were 98.24%, 89.47%, 84.16%, and 78.11%, respectively. Only IPCA achieved a recognition accuracy of over 90%, which was 8.77% lower than PCA. The recognition rate of IPCA had been significantly improved through sample partitioning and histogram equalization processing. The construction and recognition times of feature faces for IPCA, PCA, LDA, and LLE on two datasets are shown in Table 3.

**Table 3:** Four algorithms construct feature face time and recognition time on different datasets.

Algorithm	Data set	Feature face construction time (s)	Identify time (s)	Recognition accuracy (%)	References
IPCA	RenderMe-360	120	1.5	92.4	This study
PCA	RenderMe-360	240	1.7	90.8	This study
LDA	RenderMe-360	180	1.8	89.2	O. Daisuke et al. [31]
LLE	RenderMe-360	300	2.0	88.5	R. Mitchell-Heggs et al. [32]
IPCA	VoxCeleb2	250	2.0	93.1	This study
PCA	VoxCeleb2	400	2.5	91.0	This study
LDA	VoxCeleb2	350	2.2	90.5	O. Daisuke et al. [31]
LLE	VoxCeleb2	500	3.0	87.9	R. Mitchell-Heggs et al. [32]

**Fig 9:**
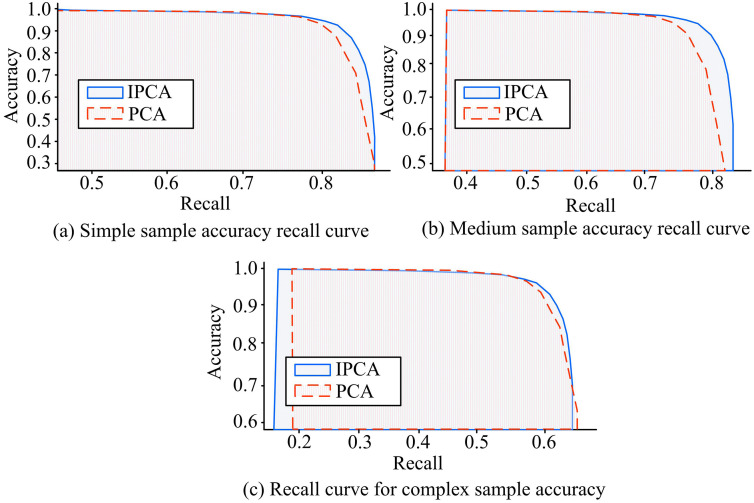
Accuracy recall rate under different samples.

In Table 3, on RenderMe-360, the IPCA constructed feature faces in 120 seconds, which was lower than the 240 seconds, 180 seconds, and 300 seconds of PCA, LDA, and LLE. The recognition time and accuracy of IPCA were 1.5s and 92.4%, which were better than the other three algorithms. On VoxCeleb2, the IPCA construction feature face time, recognition time, and recognition accuracy were 250s, 2s, and 93.1%, which were significantly superior to the other three algorithms. IPCA showed a significant time advantage on the large-scale dataset VoxCeleb2, with significantly reduced feature face construction time. IPCA also had good recognition performance in terms of recognition time and accuracy, and it was more flexible in handling incremental data, saving a lot of computing resources. In Fig 9, in the VoxCeleb2 dataset, whether the face image was classified as simple, medium, and complex difficulty, and the accuracy and recall of IPCA and IPCA were compared.

In Fig 9, under different FR difficulties, the recall rate of IPCA was superior to PCA, and the accuracy was inversely proportional to the recall rate. In Fig 9(a), under the condition of a simple dataset, when the recall rate of IPCA’s FR model was 0.85, the accuracy decreased exponentially and increased by 0.08 compared to PCA’s recall rate; In Fig 9(b) and 9(c), as the difficulty of the dataset increased, the accuracy of the FR model of IPCA decreased at recall rates of 0.74 and 0.56, respectively, but both were better than PCA, indicating that the recognition accuracy of IPCA had been improved.

### 3.2 Performance evaluation of AVS-FR model with IPCA/K-L transformation

Firstly, the recognition accuracy of the model was validated at different angles, and multiple facial angle images were selected from the VoxCeleb2 dataset for experimental verification. The AVS-FR model based on IPCA/K-L transformation was compared with CNN, Eigenfaces, Fisher faces based on LDA algorithm, Support Vector Machine (SVM), Haar Cascade classifier, Transformers, and Unified Embedding for Face Recognition and Clustering (FaceNet) based on deep learning. Table 4 shows the recognition accuracy of these algorithms at different facial angles.

**Table 4:** Recognition accuracy of different facial recognition models.

Identification model	Identification accuracy					Average recognition accuracy	References
	Left skewed 60° face	Left skewed 30° face	Deviation 0 ° (front face)	Right skewed 30° face	Right skewed 60° face		
CNN	78.45	85.20	93.60	85.50	77.85	84.12	This study
Eigenfaces	72.30	81.55	90.10	80.95	71.25	79.23	Z. Sun et al. [33]
Fisherfaces	74.85	82.70	91.50	82.00	73.60	80.93	K. Zhang et al. [34]
SVM	80.10	87.45	94.75	88.15	79.65	86.02	C. Y. Chang et al. [35]
Haar Cascade	65.30	75.15	88.90	76.25	64.95	74.11	K. N. Babu et al. [36]
FaceNet	82.50	88.85	96.00	89.50	81.75	87.72	P. Y. Chen et al. [37]
IPCA/K-L transformation	91.23	92.36	98.15	94.85	95.35	94.388	This study
Transformers	90.46	92.17	97.42	95.16	89.67	91.72	R. K. Yadav et al. [38]
2D-PCA	75.10	80.25	88.50	76.80	70.75	78.67	P. Mannocci et al. [17]
Block-PCA	77.30	820	90.00	79.25	73.40	80.03	P. Mannocci et al. [17]
DCT-PCA	74.85	81.90	89.80	78.10	72.60	79.86	P. Mannocci et al. [17]

Table 4 shows the recognition accuracy of various face recognition models at different facial angles. Among them, the AVS-FR model based on IPCA/K-L transformation performs particularly outstandingly, with an average recognition accuracy as high as 94.388%. This model performs exceptionally well from all angles. The recognition accuracy rates for left-leaning 60 degrees, left-leaning 30 degrees, frontal view, right-leaning 30 degrees, and right-leaning 60 degrees are 91.23%, 92.36%, 98.15%, 94.85%, and 95.35% respectively. Compared with other models, IPCA/K-L demonstrates outstanding robustness and reliability when dealing with complex scenarios.

In the traditional model, the average recognition accuracy of the convolutional neural network is 84.12%. Although there is an improvement in frontal recognition, the overall performance is still inferior to that of IPCA/K-L. The average recognition accuracy of Eigenfaces and Fisherfaces did not exceed 81%, indicating the limitations of these traditional methods in multi-angle face recognition. Support vector machines perform slightly better in this aspect, with an average recognition accuracy rate of 86.02%, but still do not reach the level of IPCA/K-L.

Among deep learning models, FaceNet performs the best, with an accuracy rate of up to 96% for frontal recognition, but its performance in other angles is still inferior to that of IPCA/K-L. The Transformers model performs relatively stably from different angles, especially achieving an accuracy of 97.42% in frontal recognition. However, the performance of IPCA/K-L exceeds 90% at all angles, demonstrating its advantages when dealing with various complex Angle variations.

Further comparison of 2D-PCA, Block-PCA and DCT-PCA revealed that their recognition accuracy rates were all lower than that of IPCA/K-L. The average recognition accuracy rate of 2D-PCA is 78.67%, that of Block-PCA is 80.03%, and that of DCT-PCA is 79.86%. This indicates that although these traditional PCA variants still have certain practicality in some scenarios, their performance is significantly insufficient under complex changes in facial angles. Overall, the IPCA/K-L transformation significantly enhances the accuracy of face recognition by improving feature extraction and reducing redundancy, providing a more effective technical solution for vending systems.

**Fig 10:**
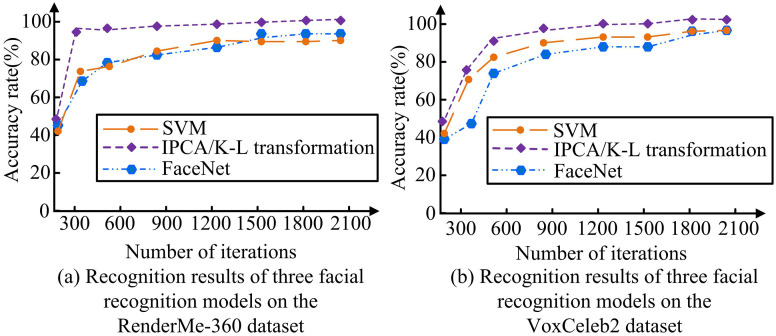
Recognition results of three facial recognition models on different datasets.

In Fig 10, SVM and FaceNet were selected for iterative experiments on the VoxCeleb2 dataset and the RenderMe-360 dataset.

**Fig 11:**
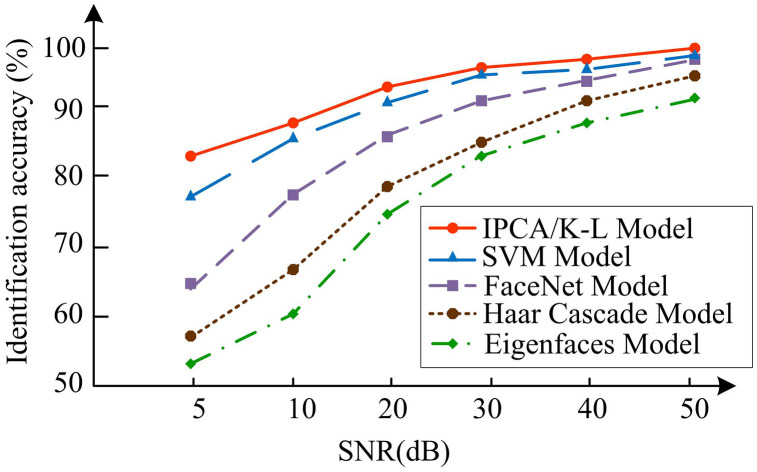
Noise tolerance data of SVM, FaceNet, IPCA/K-L transform, haar cascade, and eigenfaces.

In Fig 10(a), when the iterations reached 2100, IPCA/K-L had the highest recognition accuracy, with a recognition accuracy of 97.54%, and SVM had the best recognition accuracy. And when the iterations were 300, the accuracy of IPCA/K-L approached its maximum value, and the iteration speed was faster. In Fig 10(b), the recognition accuracy of IPCA/K-L remained the highest at different iteration times. And at 600 iterations, the recognition accuracy of IPCA/K-L on the face dataset was 91.23%, approaching the maximum value. At 2100 iterations, the recognition accuracy of IPCA/K-L, SVM, and FaceNet on VoxCeleb2 were 98.63%, 91.18%, and 92.54%, which was 7.45% and 6.09% higher than SVM and FaceNet, respectively. The IPAC recognition model introduced with K-L transform outperformed other models in recognition accuracy and iteration speed, verifying its feasibility and applicability in AVS-FR. In Fig 11, IPCA/K-L was subjected to noise resistance testing.

In Fig 11, IPCA/K-L had better noise tolerance than other models in different noise environments. In a high noise environment (5dB), the recognition accuracy of IPCA/K-L was 84.65%; In a low-noise environment (50dB), the recognition accuracy of IPCA/K-L was 97.85%. The performance of IPCA/K-L was relatively stable in various noisy environments, especially demonstrating good robustness in high noise environments. Other models had recognition accuracy below 80% in high noise environments, and the trend of curve changes was significant in different noise environments, indicating that these models were easily affected by image noise, which led to a decrease in accuracy and speed when recognizing user information. The superiority of the IPCA/K-L transformation model in AVS was verified, especially its robustness in complex noise environments. At the end of the study, the effect of face detection under different lighting conditions was verified, and the results were shown in Table 5.

**Table 5:** Face detection effect under different lighting conditions.

Light condition	Model	Recognition accuracy(%)
Low light (0–200 Lux)	IPCA/K-L transformation	85.22
Medium illumination (200–800 Lux)	IPCA/K-L transformation	94.10
High light (Above 800 Lux)	IPCA/K-L transformation	89.45
Low light (0–200 Lux)	PCA	78.67
Medium illumination (200–800 Lux)	PCA	84.30
High light (Above 800 Lux)	PCA	82.90

Under different illumination conditions, the recognition performance of IPCA/K-L transformation model shows strong robustness and adaptability, especially under medium illumination conditions, reaching a high accuracy of 94.10%. This shows that the model can effectively capture the feature information under good lighting conditions, so as to achieve efficient user identification. Under high illumination conditions, the recognition accuracy of IPCA/K-L model decreased to 89.45%, which may be due to facial shadow and reflection interference caused by strong illumination, which affected the feature extraction process. Nevertheless, IPCA/K-L maintained a high accuracy rate, much higher than that of PCA models under the same conditions. Therefore, it can be considered that IPCA/K-L model has relatively good stability when dealing with light changes. At the end of the study, the power consumption, running time and running efficiency of each method in the system are compared, and the results are shown in Table 6.

**Table 6:** Comparison of power consumption of each method in the system.

Method	Power consumption (W)	Running time (s)	Processing efficiency(FPS)	References
PCA	2.5	1.2	25	This study
LDA	3.2	1.5	20	R. Mitchell-Heggs et al. [32]
LLE	4.0	2.0	15	Z. Sun et al. [33]
CNN	6.5	3.5	10	K. Zhang et al. [34]
SVM	5.0	2.5	12	C. Y. Chang et al. [35]
IPCA/K-L	2.8	1.8	22	This study

The results in Table 6 show that the proposed method based on IPCA/K-L transformation achieves a good balance between power consumption, running time and processing efficiency. Its power consumption is 2.8W, running time is 1.8s, and processing efficiency reaches 22FPS. This performance is superior to SVM-based and CNN methods, which have significantly higher power consumption and runtime of 5.0 watts and 6.5 watts, respectively, while having lower processing efficiency.

The performance comparison results between the research and the current mainstream facial recognition methods are shown in Table 7.

**Table 7:** Comparison of power consumption of each method in the system.

Method	Model Parameters (MB)	Processing speed (FPS)	Memory consumption (MB)	Low-light robustness(%)
IPCA/K-L	0.8	58	50	84.7
ArcFace-R100	248.5	9	780	76.4
CosFace	235.2	11	750	74.9
MagFace	253.6	8	820	78.1
ElasticFace	240.1	10	760	73.5
MobileFaceNet (FP32)	4.2	45	110	82.3
MobileFaceNet (INT8)	1.1	62	30	78.9
FaceNet (Edge-TPU)	16.5	52	90	80.6

The results in Table 7 show that the IPCA/K-L method has significant advantages in model lightweight and processing efficiency. Compared with the mainstream deep models ArcFace-R100 and CosFace, its model parameters are reduced by more than 300 times and the processing speed is increased by 5–6 times. Meanwhile, the memory consumption is only 50MB, which is far lower than the over 750MB level of deep methods. Although the speed of the MobileFaceNet-INT8 version is slightly higher than that of IPCA/K-L, its recognition accuracy in low-light environments drops to 78.9%, while IPCA/K-L still maintains a robustness of 84.7%. Furthermore, IPCA/K-L can achieve a speed close to that of the Edge-TPU optimization model without relying on dedicated hardware acceleration, and the comprehensive resource consumption is more balanced.

## 4. Discussion and conclusion

The effectiveness of the AVS-FR model with IPCA/K-L transformation was verified through experimental analysis. After sample partitioning and histogram equalization processing, IPCA had significantly improved recognition rate compared to PCA, and effectively captured feature information in the image, making it more accurate for portrait recognition collected in AVS. In addition, to address the limitations of AVS-FR in dealing with complex nonlinear features in images, the K-L transform was introduced to reduce redundant information in facial data, effectively improving the recognition accuracy and processing speed of AVS in complex environments.

To improve the accuracy and processing speed of AVS-FR, enhance user experience and security, an AVS-FR model with IPCA/K-L transformation was constructed. The loss value of IPCA on RenderMe-360 tended to 3% after 60 iterations, with an average accuracy of 97%, and the algorithm terminated iterations at appropriate positions in a timely manner. When the number of feature vectors was 40, the recognition rate of IPCA was 98.24%, which was 8.77% lower than the recognition accuracy of PCA. After sample partitioning and histogram equalization, the recognition rate of IPCA had significantly improved compared to PCA. On RenderMe-360, IPCA took 120 seconds to construct feature faces, which was lower than PCA, LDA, and LLE’s 240 seconds, 180 seconds, and 300 seconds. In addition, the recognition time and accuracy of IPCA were 1.5s and 92.4%, which were better than the other three algorithms, proving that the improved IPCA recognition accuracy had been improved. The average recognition accuracy of IPCA/K-L at various angles of FR was 94.388%. When the iterations reached 2100, IPCA/K-L had the highest recognition accuracy, with a recognition accuracy of 97.54%, proving the effectiveness of the model. Under different lighting conditions, the recognition accuracy of the model based on IPCA/K-L transformation is 85.22% under low lighting conditions, while it is significantly increased to 94.10% under medium lighting conditions, indicating that it can effectively capture feature information under ideal lighting conditions to ensure efficient user recognition. In summary, IPCA/K-L can be effectively applied to AVS, which is of great significance for improving user experience and system security. The limitation of this study is that it can limit the real-time performance and computational efficiency of the algorithm when computer resources are limited. Future research will consider combining hardware acceleration technology to enhance model on the system.

## Supporting information

S1 File Minimal data set definition. (DOCX)
