# Supplementary material for: PCA/K-L transformation facial recognition method for vending systems
Source: PLoS One. 2025 Dec 10;20(12):e0336225. doi: 10.1371/journal.pone.0336225 (PMC12694864; doi:10.1371/journal.pone.0336225)
Supplement: S1 File — (DOCX) [file pone.0336225.s001.docx]

Figure7(a) Acc Epoch Loss Curve of IPCA Algorithm on RenderMe-360 Dataset

| Indicator | Number of iterations | | | | |
| --- | --- | --- | --- | --- | --- |
|  | 10 | 20 | 30 | 40 | 50 |
| Train-acc | 0.88 | 0.90 | 0.92 | 0.94 | 0.97 |
| Train-loss | 0.37 | 0.22 | 0.12 | 0.07 | 0.03 |
| val-acc | 0.90 | 0.91 | 0.93 | 0.95 | 0.97 |
| val-loss | 0.26 | 0.20 | 0.11 | 0.06 | 0.03 |

Figure7(b) Acc Epoch Loss Curve of IPCA Algorithm on VoxCeleb2 Dataset

| Indicator | Number of iterations | | | | |
| --- | --- | --- | --- | --- | --- |
|  | 10 | 20 | 30 | 40 | 50 |
| Train-acc | 0.62 | 0.75 | 0.80 | 0.84 | 0.85 |
| Train-loss | 0.25 | 0.26 | 0.25 | 0.25 | 0.25 |
| val-acc | 0.75 | 0.80 | 0.84 | 0.84 | 0.84 |
| val-loss | 0.25 | 0.24 | 0.24 | 0.23 | 0.23 |

Figure8(a) Acc Epoch Loss Curve of IPCA Algorithm on VoxCeleb2 Dataset

| Number of iterations | Model | | | |
| --- | --- | --- | --- | --- |
|  | IPCA | PCA | LDA | LLE |
| 4 | 0.6534 | 0.5312 | 0.4223 | 0.3912 |
| 8 | 0.7345 | 0.6248 | 0.5647 | 0.3945 |
| 12 | 0.7964 | 0.6978 | 0.7204 | 0.5218 |
| 16 | 0.8675 | 0.7216 | 0.7007 | 0.6677 |
| 20 | 0.8923 | 0.7268 | 0.7712 | 0.6502 |
| 24 | 0.9241 | 0.7628 | 0.8313 | 0.7258 |
| 28 | 0.9254 | 0.7911 | 0.8788 | 0.7513 |
| 32 | 0.9523 | 0.8042 | 0.8256 | 0.7546 |
| 36 | 0.9525 | 0.8405 | 0.8549 | 0.7735 |
| 40 | 0.9632 | 0.8426 | 0.9034 | 0.7749 |

Figure8(b) Recognition rate results of different algorithms on the VoxCeleb2 dataset

| Number of iterations | Model | | | |
| --- | --- | --- | --- | --- |
|  | IPCA | PCA | LDA | LLE |
| 4 | 0.6000 | 0.5232 | 0.4536 | 0.4524 |
| 8 | 0.6753 | 0.5381 | 0.4690 | 0.4607 |
| 12 | 0.7245 | 0.5904 | 0.4956 | 0.4902 |
| 16 | 0.7890 | 0.6123 | 0.5321 | 0.5109 |
| 20 | 0.8301 | 0.7184 | 0.5789 | 0.5562 |
| 24 | 0.8650 | 0.7540 | 0.6102 | 0.5998 |
| 28 | 0.9012 | 0.7856 | 0.6408 | 0.6300 |
| 32 | 0.9523 | 0.8045 | 0.7001 | 0.6705 |
| 36 | 0.9824 | 0.8947 | 0.8416 | 0.7811 |
| 40 | 0.9824 | 0.8947 | 0.8416 | 0.7811 |

Figure 9(a) Simple sample accuracy recall curve

| Model | Recall | | | |
| --- | --- | --- | --- | --- |
|  | 0.5 | 0.6 | 0.7 | 0.8 |
| IPCA Acc | 0.99 | 0.98 | 0.97 | 0.95 |
| PCA Acc | 0.99 | 0.98 | 0.96 | 0.94 |

Figure 9(b) Simple sample accuracy recall curve

| Model | Recall | | | |
| --- | --- | --- | --- | --- |
|  | 0.5 | 0.6 | 0.7 | 0.8 |
| IPCA Acc | 0.99 | 0.98 | 0.97 | 0.90 |
| PCA Acc | 0.99 | 0.98 | 0.96 | 0.70 |

Figure 9(c) Simple sample accuracy recall curve

| Model | Recall | | | |
| --- | --- | --- | --- | --- |
|  | 0.3 | 0.4 | 0.5 | 0.6 |
| IPCA Acc | 0.99 | 0.98 | 0.97 | 0.93 |
| PCA Acc | 0.99 | 0.97 | 0.96 | 0.90 |

Figure10(a) Recognition results of three facial recognition models on the RenderMe-360 dataset

| Model | | SVM | IPCA/K-L | FaceNet |
| --- | --- | --- | --- | --- |
| Number of iterations | 300 | 0.7252 | 0.9024 | 0.7024 |
|  | 600 | 0.7553 | 0.9257 | 0.7389 |
|  | 900 | 0.7856 | 0.9431 | 0.7495 |
|  | 1200 | 0.8122 | 0.9478 | 0.7598 |
|  | 1500 | 0.8321 | 0.9600 | 0.7714 |
|  | 1800 | 0.8467 | 0.9683 | 0.7780 |
|  | 2100 | 0.8541 | 0.9754 | 0.8827 |

Figure10(b) Recognition results of three facial recognition models on the RenderMe-360 dataset

| Model | | SVM | IPCA/K-L | FaceNet |
| --- | --- | --- | --- | --- |
| Number of iterations | 300 | 0.7154 | 0.7853 | 0.4325 |
|  | 600 | 0.7492 | 0.9121 | 0.4956 |
|  | 900 | 0.8101 | 0.9478 | 0.5304 |
|  | 1200 | 0.8603 | 0.9705 | 0.6121 |
|  | 1500 | 0.8709 | 0.9734 | 0.7702 |
|  | 1800 | 0.8845 | 0.9850 | 0.7908 |
|  | 2100 | 0.9118 | 0.9863 | 0.8254 |

Figure 11 Noise tolerance data of SVM, FaceNet, IPCA/K-L transform, haar cascade, and eigenfaces

| Model | | IPCA/K-L | SVM | FaceNet | Haar Cascade | Eigenfaces |
| --- | --- | --- | --- | --- | --- | --- |
| SNR(dB) | 5 | 0.8465 | 0.7759 | 0.6514 | 0.5674 | 0.5311 |
|  | 10 | 0.8600 | 0.7880 | 0.6703 | 0.5800 | 0.5502 |
|  | 20 | 0.8902 | 0.8045 | 0.7057 | 0.6301 | 0.5803 |
|  | 30 | 0.9101 | 0.8300 | 0.7405 | 0.6707 | 0.6134 |
|  | 40 | 0.9436 | 0.8504 | 0.7801 | 0.7513 | 0.7009 |
|  | 50 | 0.9785 | 0.9647 | 0.9601 | 0.9512 | 0.9145 |
